# Animal reuse in safety assessment studies: a review of current practices, challenges and 3Rs opportunities

**DOI:** 10.3389/ftox.2026.1854281

**Published:** 2026-09-14

**Authors:** John S. Kendrick, David A. Shaw, Marie Jourdain, Claudia Senn, Helen Beyer, Fiona R. Sewell

**Affiliations:** 1 Labcorp, Harrogate, United Kingdom; 2 Roche Pharma Research and Early Development, Roche Innovation Center, Basel, Switzerland; 3 Silabe-Université de Strasbourg, Niederhausbergen, France; 4 National Centre for the Replacement, Refinement and Reduction of Animals in Research (NC3Rs), London, United Kingdom

**Keywords:** 3Rs, *in vivo*, NAMs, nonclinical, non-naïve, reuse, sustainability, toxicology

## Abstract

Regulatory nonclinical safety assessment in animals remains an essential part of the drug development process. Historically, naive animals are used within most nonclinical studies to avoid confounding variables such as prior drug exposure. However, opportunities exist to advance the 3Rs (Replacement, Reduction and Refinement) by reusing animals in studies when scientifically and ethically justified, without compromising scientific integrity or animal welfare or exceeding permitted cumulative physiological and psychological burden. Reuse is already standard practice in pharmacokinetic (PK) and safety pharmacology studies, yet its application in toxicology remains limited. This review explores how broader adoption of reuse as a 3Rs strategy can reduce overall animal numbers, while ensuring reuse does not compromise animal welfare or the scientific integrity of the study. Animal reuse can avoid and reduce known stressors prior to study start, such as transportation, quarantine and acclimation to new environments, by allowing animals to remain in a familiar environment for longer periods extending the time for positive reinforcement training and to establish group hierarchy. Appropriate reuse supports the sustainability and ethical use of animal models. Challenges of reuse include managing the cumulative lifetime burden of the animal, ensuring adequate washout and recovery periods, and addressing scientific concerns such as immunogenicity risks associated with developing anti-drug antibodies (ADA) against biologics or pre-existing immunity to gene therapies and the rising frequency of age-related background lesions. We propose a tiered framework to guide reuse decisions: Tier 1 includes studies where reuse is already standard and achievable; Tier 2 covers studies where reuse is generally feasible with appropriate scientific and ethical rationale; and Tier 3 includes studies where reuse is more challenging due to regulatory, scientific or welfare considerations, although enrolling non-naive animals at study start may still be possible without compromising data interpretation. Enabling wider implementation of reuse will require industry-wide collaboration, standardized decision tools, and proactive engagement with contract research organizations. When applied appropriately, reuse can reduce overall animal use while maintaining robust safety assessment. In parallel with the evolving adoption of new approach methodologies, reuse offers an additional practical avenue to support a more sustainable and humane nonclinical testing paradigm.

## 1 Introduction

Nonclinical *in vivo* safety assessment is an integral, regulatory-required component of pharmaceutical development. Conducting *in vivo* safety pharmacology and toxicology studies is important to evaluate new therapies for potential on- and off-target effects and to establish a no observed adverse effect level (NOAEL). Gaining an understanding of exposure kinetics and the reversibility of any adverse effects are key to establishing a safe starting dose for first-in-human clinical trials and to support safety assessment throughout later stages of clinical development. These nonclinical *in vivo* studies represent a significant proportion of the safety assessment program for both large and small molecule drug candidates (Shen et al., 2019; Stark and Steger-Hartmann, 2016).

Over the course of the last decade, the nonclinical safety assessment field has embraced the challenge of increasing implementation of the 3Rs (reduce, refine, replace) whenever scientifically possible (Chapman et al., 2014; Chien et al., 2023; Hasselgren et al., 2019; Luijten et al., 2020; Prior et al., 2023; Prior et al., 2019).

Perspectives on animal reuse are often based on individual experiences, societal norms, and regulatory frameworks. In some regions ethical guidance has been formalized into law, such as the UK Animals (Scientific Procedures) Act (1986) (ASPA, 1986) and the EU Directive 2010/63 (EU, 2010). Conducting scientific research involving non-naive (i.e., previously treated/tested) animals requires careful consideration, balancing the overall experience of the animal against the cumulative harm of the regulated procedures the animal undergoes. The impact of study procedures, depending on their type, duration, and frequency, in combination with any pharmacological or toxicological effects seen in the animal due to the administered compound, will affect the overall assessment.

The overall “lifetime” experience of the animal is not only a consequence of the scientific investigations to which it is subject. Generally, animals obtained from approved suppliers need to be transported to locations where they will be used within scientific studies. In the case of non-rodent species, such as minipig, dog, and non-human primate (NHP), they may require international transit, and it is therefore necessary to consider the impact of long transportation journeys on the overall welfare of naive animals.

Although the development and regulatory uptake of new approach methodologies (NAMs) continue to grow (Avila et al., 2023; Sewell et al., 2024). Regulatory nonclinical studies in animals remain essential in most regions, in line with current international guidance (e.g., International Council for Harmonisation of Technical Requirements for Pharmaceuticals for Human Use (ICH) guidelines). In the short to medium term, NAMs and *in vivo* studies will continue to be used in combination; therefore, identifying opportunities to refine or reduce animal use - such as appropriate reuse - remains important. When implemented appropriately, reuse provides a 3Rs-aligned approach to reduce the number of naive animals entering the system while ensuring that the cumulative burden is carefully managed.

This review is primarily conceptual and aims to support greater awareness of the opportunities and challenges associated with the reuse of animals beyond established areas such as pharmacology and safety pharmacology. We acknowledge the current limited availability of systematically collated empirical datasets on animal reuse, particularly within regulatory toxicology, reflecting the historical lack of coordinated data capture across companies. Where possible, we draw on published literature, industry experience, and a worked case study (Supplementary Appendix 1) to illustrate implementation considerations and practical outcomes.

## 2 Rationale for reuse

### 2.1 Ethical and welfare considerations

As a key strategy for achieving a 3Rs impact, animal reuse involves the scientifically and ethically justified use of the same animal in more than one experiment or procedure. While the need for nonclinical *in vivo* studies remains, increased animal reuse, beyond current industry norms, will help reduce the total number of animals bred for scientific research and will refine *in vivo* study conduct.

Reuse is still “use” but can reduce the need for more naive animals to enter the supply chain and allows for those animals already in test facilities to age and become more accustomed and conditioned to their environment, maximizing the contribution that each animal provides to scientific research. These benefits must be weighed on a case-by-case basis against the long-term detrimental effects of long-term captivity on animal behavior, and the practical difficulties involved in the handling and long-term housing of larger species.

Reuse has been an established practice for specific study types for many years, such as *in vivo* pharmacokinetic screening studies and safety pharmacology studies in non-rodents, where single or repeat low-dose administrations allow measurement of systemic concentrations, ECGs and metabolites (Table 1). In these specific studies, there is no expected adverse impact of the drug on the animal, and the administered drug can be washed-out prior to the animal’s participation in future studies. Any observed clinical signs are more likely attributable to stress, due to dosing and sampling procedures, rather than toxicity. A veterinary professional must still assess the potential reuse of any animal to ensure it is healthy and viable and has not exceeded its lifetime cumulative physiological burden prior to enrollment in future studies. This burden is the total impact of all procedures, stressors, and experiences an animal has endured over the course of its life. This assessment includes evaluating the impact of past procedures, the effects of previous substances, the frequency of handling, psychological stress from transport or housing changes, and the adequacy of recovery periods.

**TABLE 1:** Typical industry and CRO standards: conditions and operational limits for animal reuse.

Species	Max studies/experiments	Key conditions and procedures
Mice and rats: General	Max 2 studies/phases (e.g., IV/PO crossover)	Restricted to small molecules Requires 1–2-week recoveryMicro-sampling (<30 µL) is preferred to enable full PK/PD profiling in single animals
Rats: bile-duct cannulation	Max 6 investigations over 28 days	2-day minimum washout between uses Bile is recirculated via a pin-port system during group-housing to maintain physiological health
Mice and rats: CNS cannulation	Rats: 6 studies/6 weeks Mice: 4 studies/4 weeks	2-day minimum washout CNS cannulation is prioritized for serial CSF sampling to reduce total animal numbers
Rats: jugular vein catheter	Max 2 studies	Both animal and catheter patency must be confirmed healthy A mandatory 2-week recovery is required between studies
Rabbits	Max 10 studies over 2 years	Minimum 2-week recovery between dosing/bleeding phases Single-use animals may be transferred to other non-terminal programs
NHP (cynomolgus macaques)	2–3-week washout between studies	Colony: up to 12 PK studies/year (10–12-year lifespan) CRO: Typically, 2-year utilization. Includes mandatory ADA monitoring; positive animals are excluded from future biologic testing
Dogs	1–2-week washout between studies	2-year utilization limit at CROs Surgically cannulated or telemetered dogs may be reused for up to 12 months pending catheter patency
Minipigs	1–2-week washout between experiments	Euthanized if >1 year old or weight exceeds 20–26 kg. Respite periods are adjusted based on sampling method (needle-stick vs. surgical cannula)
Clearance period definitions ◦ Recovery: Animal must return to a healthy, “naive equivalent” state before subsequent studies ◦ Washout: Time required for complete elimination or metabolism of prior test items Scientific integrity ◦ ADA monitoring is mandatory for biologics in NHPs; ADA-positive animals are excluded from future large-molecule studies Health clearance ◦ Prior to reassignment, animals must meet baseline liver (DILI) and kidney biomarker criteria (e.g., ALT, GLDH, KIM-1)		

### 2.2 Pharmacological and biological considerations

In most nonclinical *in vivo* safety assessment study types, such as general repeat dose toxicity studies, the default approach is to enroll naive animals. This reflects the routine inclusion of histopathology, where background lesions and/or chronic lesions (induced by previous drug application) would affect the interpretation of test-item related lesions of the current study, and the need to minimize variability by using animals that are homogeneous in age and bodyweight and free from any previous drug effects. To achieve animal reuse, a “washout” period is therefore essential. This should allow for complete drug clearance and the resolution of any prior test-item effects (for a new biological or chemical entity for instance). This step mitigates the risk of drug-drug interactions and prevents cumulative toxicity if both agents affect the same target. Washout periods, in the case of biologics with long half-life exposures, can be lengthy and logistically challenging to return the animal to its physiological baseline. Additionally, the production and presence of clearing or neutralizing ADA following exposure to biological drugs can potentially negate exposure of the subsequent administered (biological) drug. The presence of ADA does not always lead to adverse consequences following the administration of a second biological drug, and it is highly unlikely to affect the pharmacokinetics of a subsequent small molecule drug (see Section 5.1).

Despite the valid risks, concerns and challenges of increasing reuse (when implemented in an appropriate and case-by-case manner), it may help address several issues facing the pharmaceutical industry, such as sustainability, ethical use of animals and in some contexts improved scientific relevance to humans through enhanced translatability (see Section 4.2).

Reuse contributes to an integrated 3Rs approach that balances the welfare burden and lifetime experience of animals. New draft guidance from the FDA (Oncology Pharmaceuticals: Streamlined Nonclinical Safety Studies for Biologics and Conjugated Products Guidance for Industry, distributed in May 2026) highlights that Sponsors could propose alternative approaches to support the 3-month general toxicology study, such as a non-terminal toxicology study. Non-terminal studies may allow the cohort of animals from the 3-month Toxicity study to be reused for other research after drug clearance and veterinary assessment.

### 2.3 A proposed tiered framework for reuse decisions

To address the scientific, ethical, and operational challenges associated with reuse, we propose a tiered strategy to guide decision-making. Within this strategic approach, we propose three tiers that stratify reuse according to feasibility and inherent risks.

Tier 1 studies are investigations and study designs that already lend themselves to routine use of non-naive animals or enable the reuse of animals post-study completion, providing the opportunity to encourage reuse on a case-by-case basis. This tier contains study types where reuse is already the standard industry approach. However, reuse cannot always be achieved in all studies where reuse is standard, either due to low availability of non-naive animals or lack of a biologically non-naive PK colony.

Tier 2 study types are investigations and studies where implementation of non-naive animals into the study may be possible, and reuse at the end of the study could also be achieved to meet the regulatory scientific endpoints without euthanasia.

In Tier 3 studies, the probability of implementing reuse post study is lower (either due to a regulatory need for certain endpoints requiring euthanasia such as histopathology or high likelihood the animals will be unsuitable for reuse at the end of the study), however enrollment of non-naive animals into the study (at the start) may still be possible and would not impact study interpretation. Examples of studies into which non-naive animals could be enrolled are given in Table 2. Further reuse of the animal after enrollment into these studies will be dictated by the necessary regulatory required endpoints such as histology.

**TABLE 2:** Tiered framework for reuse potential within regulatory study types.

Tier	Reuse potential	Associated study types	Rationale for reuse
TIER 1	Standard(High)	PK/PD studies Safety pharmacology (telemetered) Radiolabeled ADME Mechanistic/Exploratory PK - no need for necropsy Control animals (from non-GLP studies)	Studies are generally non-terminal Procedures are usually low dose/low risk Recovery to “naive equivalent” status is predictable
TIER 2	Case-by-case (moderate)	MTD/DRF combinations Feasibility/pilot studies ePPND controls	Reuse achievable if no exaggerated pharmacological or tox effects observed Endpoints do not require tissue examination
TIER 3	Difficult*(Low)	GLP toxicity studies and other pivotal regulatory studies* Studies requiring histopathology Novel long-acting modalities High likelihood of ADA	Reuse is precluded by irreversible effects Long-acting genetic modifications Necessity for histopathological endpoints for health authority submissions High ADA risk

* Reuse can be achieved by enrolling non-naïve animals into terminal GLP, studies.

In Supplementary Appendix 1, a case study example outlines how a cohort of non-naive animals from a non-terminal study can facilitate reuse and how non-naive animals can be integrated into a subsequent terminal study.

## 3 Species specific considerations for reuse

Discussions on the topic of reuse in recent years have extensively focused on biologically non-naive NHPs. The IQ consortium’s (https://iqconsortium.org/) NHP reuse working group published a position paper in 2022 (Mattis et al., 2022) that was supportive of increasing reuse whilst also outlining the challenges it presents.

### 3.1 Non-human primates (NHP)

Health authorities, including the US Food and Drug Administration (FDA) and the European Medicines Agency (EMA) now discourage the use of NHPs for small molecule toxicology programs. Recent guidelines (EMA, 2025; FDA, 2025) indicate that the use of dogs or pigs is generally accepted and sufficiently relevant for these programs. For biologics, where NHPs remain the most scientifically relevant model due to target homology, reuse can mitigate supply chain issues or animal shortages (such as sexually mature animals) and significantly lower costs–a critical factor for biotech companies. However, reusing NHPs for biologics presents specific challenges, particularly regarding the long tissue half-life of these molecules and the potential for ADAs. The risk that prior ADA generation might affect the exposure of a second drug is largely theoretical, however it creates uncertainty and without prior assessment of the ADA risk it could be considered unethical to implement reuse. Assessment of ADA in biologically non-naive animals is possible with available generic assays. With IgG clearance potentially taking up to several months, strategic management and extended washout periods are essential. Despite these hurdles, several pharmaceutical companies have successfully managed biologically non-naive NHP colonies without reported adverse findings. The long lifespan of NHP (up to 30 years depending on the species) provides the opportunity for reuse across multiple studies, enhancing their potential utility.

**FIGURE 1:**
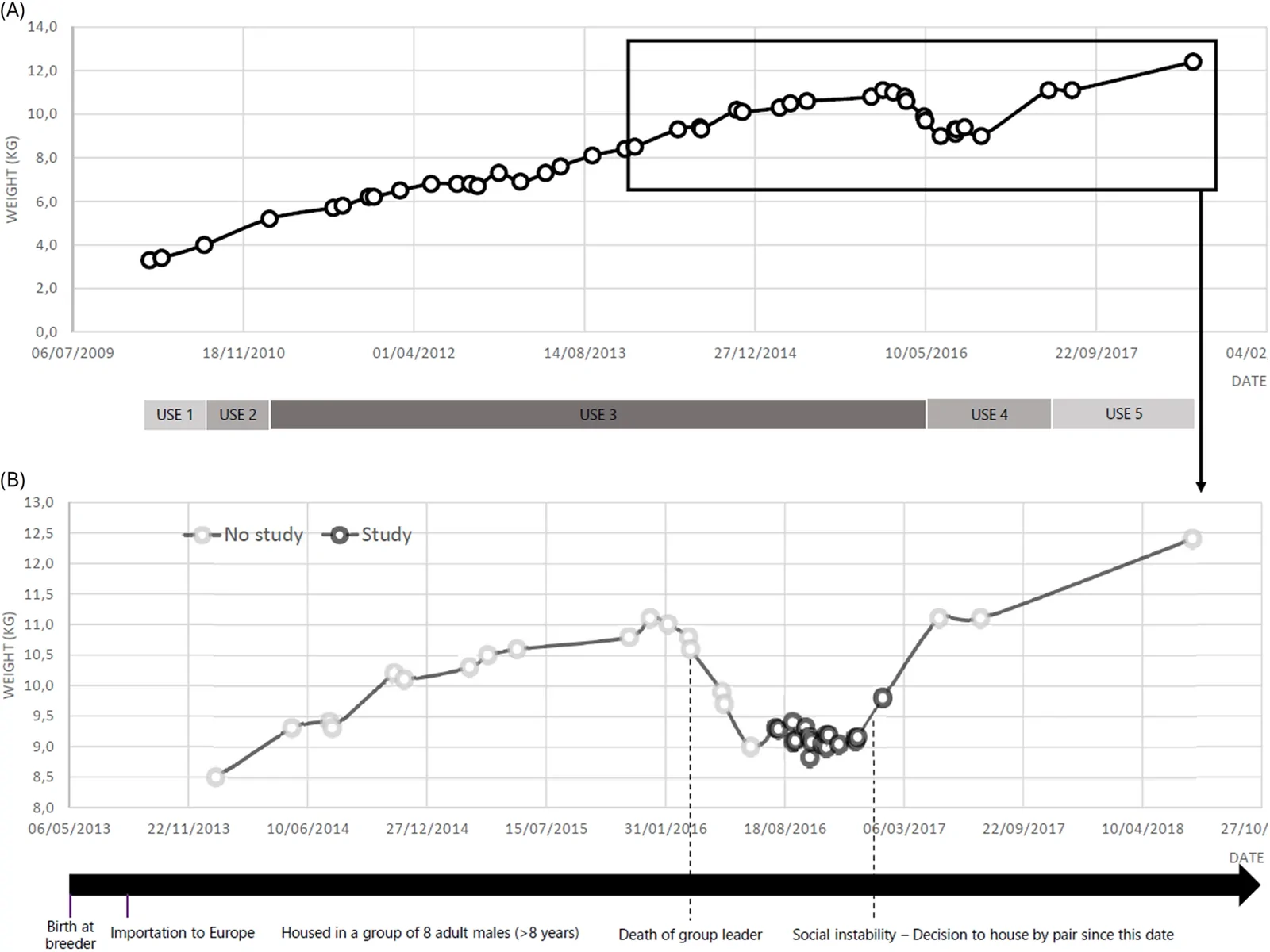
Longitudinal bodyweight fluctuations in an adult male cynomolgus macaque. **(A)** Shows the animal’s bodyweight over 10 years. **(B)** Highlights the animal’s bodyweight loss in 2016.

An example of the bodyweight variations of one cynomolgus macaque over its lifetime is presented in Figure 1. This individual animal’s bodyweight graph shows the involvement of the animal in several mild severity studies (designated as USE 1–4) over a 12-year period. The graph shows that USE 1–3 had very little impact on the animal’s bodyweight as the animal was growing into adulthood, however after an environmental change and subsequent disruption of the social group hierarchy prior to USE 4, there was an impact on the bodyweight that took some time to recover. These data illustrate the challenges of long-term housing for mature NHP, due to the need to maintain social structure. Changes in social groups may lead to instability that can have physiological (bodyweight loss) and psychological (stress) consequences.

### 3.2 Dogs

The dog (commonly the Beagle) is an accepted non-rodent species for small molecule safety assessment and offers a less complicated path for reuse than NHPs. This is largely because small molecules typically clear the body within 24–48 h and do not usually provoke lasting immunological effects, making subsequent reuse scientifically and ethically more straightforward. The dog may also be used in large molecule projects; however, its homology to human targets is not as close to humans and may not provide sufficient relevance. Colonies of dogs for PK, PK/PD and safety pharmacology studies are available at CROs globally, even permitting assessment of biological drug candidates. The management of these colonies requires regular cumulative stress assessments and careful monitoring of the health and wellbeing of the animals. Animals that reach the threshold of their physiological or psychological burden are frequently eligible for rehoming as pets or assistance animals and can adapt well to life outside animal units.

The dog supply chain faces its own pressures due to industry’s reliance on a limited number of reputable breeders. Furthermore, the global NHP shortage has forced more programs to use the dog as an alternative non-rodent species to NHP, placing an additional strain on current dog availability.

### 3.3 Minipigs

The use of the minipig is continuing to gain traction for the nonclinical safety assessment of small molecule therapeutics and their utility for biologics is also established. This growing interest is reflected in ongoing discussions within industry platforms such as the Minipig Research Forum (https://minipigresearchforum.org/) and IQ DruSafe (Clarke et al., 2024), but also in major collaborative initiative such as the Innovative health Initiative (IHI) NHPig project (https://www.ihi.europa.eu/projects-results/project-factsheets/nhpig), which aims to reduce reliance on NHPs by advancing minipig--based models for nonclinical safety assessment. The Göttingen minipig is also often viewed as an alternative to the dog, though debates persist regarding their equivalent sentience to the dog and rising status as companion animals. In some regions, such as the UK, the selection of minipig should precede the selection of dogs and needs to be ruled out first on scientific grounds to proceed with another animal model.

The main challenge with minipig reuse is their continuous growth, and many older animals reach weights that prevent their inclusion into studies due to test material limitations. Their rapid growth (up to 2 kg/month) complicates handling and significantly increases test material requirements compared to dogs or NHPs. While higher test material demands are a practical concern, species selection should ultimately be driven by scientific relevance and translatability rather than material costs.

Other logistical challenges exist with minipigs that can cause animal welfare concerns, particularly regarding blood sampling, which is more difficult in this species due to limited peripheral venous access, the need for increased restraint or sedation, and the potential for bruising or vein collapse with repeated sampling; however, improvements in the long-term patency of surgically implanted cannulas could significantly facilitate reuse for continuous infusion and repeated sampling.

### 3.4 Rodents and rabbits

The reuse potential of rodents (rats, mice, hamsters, guinea pigs) and lagomorphs (rabbits) is often underestimated. Advances in micro-sampling and bioanalytical sensitivity now allow for sufficient toxicokinetic data generation with minimal blood volume (Chapman et al., 2014). Furthermore, surgical cannulation offers a refined alternative for dosing and sampling, enabling crossover designs that directly reduce animal numbers. Within the field of safety pharmacology, the combination of cardiovascular safety studies with Irwin and respiratory assessments can be achieved using the same cohort of animals, eliminating the need for separate naive groups. Similarly, crossover PK studies can utilize reuse to explore absolute bioavailability or to repurpose control cohorts for subsequent safety assessments.

## 4 Benefits of reuse

### 4.1 Reuse can reduce the impact of stress factors in studies

By replacing naive animals for non-naive animals in nonclinical studies, a reduction in demand for naive animals is created and therefore fewer animals are required to be reproduced at breeding facilities. Reuse has other benefits that contribute to the 3Rs, in terms of increased animal welfare and study refinement. One factor that may affect the reliability of nonclinical data is stress (either acute, episodic acute or chronic), which may arise from a multitude of physical and environmental stressors in the pre-study and in-life phases such as transportation, insufficient acclimation to housing conditions and social relationship disruption. Differing training needs and extensive procedural demands can also contribute to the lifetime cumulative stress burden on the animal. These considerations apply across all species: although often discussed in the context of large animals, small animals such as rodents and rabbits are also affected by transport, housing changes, handling and procedural demand. While many of these factors can be mitigated through extended acclimation periods for naive animals; non-naive animals have already learned to adapt to these stressors, which can reduce baseline variability at study start.

The use of control animals can help differentiate observed changes due to procedural stress from test material related effects; however longer-term stressors may impact the biology of the animal on an individual basis in the same manner that humans react differently to chronic and episodic stress factors. Stress can potentially influence the data obtained if the animal’s immune system and subsequent responses are modulated. Any assessment of immunological parameters may become more complex and increase the variability of the group data set. Immunological modulation due to stress can also result in latent virus reactivation or changes to the commensal gut microflora leading to altered metabolism. Many nonclinical *in vivo* studies, especially for the safety assessment of biologics, place high value on immunological endpoints such as cytokines, immuno-phenotyping, and T-cell-dependent antibody response (TDAR) assessments. Animals suffering from acute, episodic or chronic stress will undoubtedly demonstrate a stress-related effect that will complicate or even preclude interpretation (Segerstrom and Miller, 2004), potentially compromising the main aims of the study and obscuring drug-related effects. Although the magnitude and expression of stress may vary by species and facility, acknowledging these factors alongside scientific and regulatory considerations provides a more complete context for assessing reuse; as robust, stress-free animals are paramount for achieving good scientific outcomes and generating the most translatable data sets. While good animal husbandry practices, environmental enrichment and acclimation to procedures at animal facilities can help to overcome stressors, their success depends heavily on the rigor and consistency of these procedures, as well as the specific housing conditions, which may vary geographically.

While longer acclimation, extended training, and reduced procedures can lower stress for naive animals, the reality of demands for accelerated drug development often makes slower study cycles impractical. Animal reuse can work within this fast-paced paradigm by achieving the same goal more efficiently. Because non-naive animals are already at the facility, they are fully accustomed to standard procedures and routines. This approach eliminates the transportation-related stress that would occur in the weeks before a new study. These animals will have been acclimated to the test site for a significantly longer duration than newly arrived subjects, meaning any stress from their initial transport should have long since dissipated.

Animals considered suitable for reuse must first complete a washout period (and possibly an extended recovery) before passing a full veterinary health check. Recovery refers to a period where the animal returns to a state of good health equivalent to a naive animal and is deemed ready for a subsequent study without the cumulative burden exceeding the maximum permitted by the experimentation license, local regulations or ethical review. Washout, by contrast, is a period when the test item administered in the previous study (and its metabolites) is no longer present in the animals’ system (i.e., eliminated or fully metabolized). The potential for prolonged physiological changes due to chronic stress, whether from previous studies or disrupted social relationships, must still be considered. In addition to reducing stress factors by using better-acclimated animals, reuse also alleviates the need for further breeding and removes the distress associated with transporting new animals through the supply chain.

Most research establishments have robust, veterinary approved welfare procedures for the acclimation and training of naive animals post-arrival. The duration of the acclimation period can vary depending on the species, the type of study, and the training requirements. As acclimation is progressive and leads to ultimate adaptation to the new environment, it is an underpinning aspect of the use of animals in scientific research. However, both prolonged periods of inactivity (leading to boredom) and excessive regular stimulation through use (leading to over-stimulation) can cause stress and atypical behavior.

Enabling regular reuse, if appropriate, provides non-naive animals already *in situ* with a more sustained and balanced acclimation period, as well as greater familiarity with their environment, animal care staff, and routine husbandry practices. This includes consistent daily procedures and standardized dietary supplementation, all of which help reduce stress levels and improve the animals’ readiness and training for study procedures. This in turn provides greater normality of baseline physiological parameters, which form part of the standard clinical pathology and pharmacodynamics assessments following drug administration. Inevitably, achieving lower stress in animals allocated to a study promotes a more reliable scientific baseline to interpret scientific data and treatment-related effects (Janssens et al., 2021; Stuart and Robinson, 2015). Within European testing facilities, animals are provided with some of the largest and enriched environments in the pharmaceutical industry (ASPA, 1986; EU, 2010). EU housing minimum standards are often exceeded (e.g., see (Baker et al., 2026) in relation to NHPs specifically), and animals are group-housed unless the scientific endpoint demands a short duration of single housing. Socialization with caretakers for periods of play, training, and sham dosing are normally provided multiple times per week during the acclimation and pre-dose phase. Returning animals after completion of the study to “stock” housing that matches the same standards is essential as well as the same level of pre-study enrichment. The quality of life of the animal in the unit, when no longer involved in study procedures, is an equally important factor in the reuse assessment.

### 4.2 Scientific benefits of reuse: improving the predictive value of preclinical studies

The reliability and translatability of nonclinical *in vivo* safety data are under widespread scrutiny, highlighting a need to better understand the limitations of data from standard regulatory studies (Clark and Steger-Hartmann, 2018; Leach et al., 2024). Nonclinical *in vivo* safety studies have traditionally used healthy, homogeneous animal populations.

While this approach was intended to maximize statistical power by reducing baseline variability, it is also a recognized limitation, as it fails to capture the genetic and physiological diversity that influences drug responses in the polymorphic human population. Because reused animals often come from different litters, suppliers, age ranges or previous study backgrounds; reuse naturally results in more heterogeneous study cohorts than those assembled from newly procured naive animals. However, increased heterogeneity may also introduce additional variability that can complicate data interpretation, especially in small toxicology group sizes, and may in some cases reduce sensitivity to detect subtle treatment-related effects (Richter et al., 2009).

Using a more heterogeneous mix of animals (such as naive and non-naive, or those with wider age and weight ranges) could help bridge this translational gap. However, this approach is only viable if the use of alternative tools and technologies, such as multi-omics endpoints and genetics or phenotypic screening, provides the necessary supportive data. Rather than a design weakness, this increased variability can in some contexts better reflect the diversity of human clinical trials and the intended patient population, potentially enhancing translatability and predictivity and refining mechanistic understanding of drug safety. The extent to which this improves translational relevance depends on the study context, species, and endpoints measured, and is therefore not universally advantageous across all toxicology study types (Voelkl et al., 2020; Voelkl et al., 2018).

The traditional justifications for homogeneity are also weakening. While homogeneity has been prioritized to establish NOAELs or clear dose-response relationships, these measures are not always necessary. For biologics, confirming the drug interacts with its intended target is often more important than defining a detailed dose–response curve. Dose-range finding studies likewise rely on observing some toxicity to establish appropriate dose levels. Nonetheless, for many regulatory study types, maintaining controlled variability remains important to ensure clear identification of dose–response relationships and safety margins, and any move toward more heterogeneous cohorts must be balanced against regulatory expectations (Richter et al., 2009).

Furthermore, the statistical arguments for homogeneity within small cohorts (typically used per group in safety studies) are increasingly challenged. Standard regulatory studies are “snapshot” assessments, not powered by statistical significance. In practice, regulators and scientists increasingly find that the biological context of individual data points is often more meaningful than any statistically significant group mean (Lazar, 2016). Shifting the focus towards individual responses relative to baseline may provide a more meaningful understanding of test item effects, particularly where small group sizes can otherwise obscure true signals or generate false negatives. This does not negate the utility of homogeneous cohorts for certain regulatory endpoints but highlights that both approaches have strengths and limitations that must be weighed case-by-case (Wurbel, 2000).

The move away from established norms represents a substantial behavioral shift. The key challenge lies in building confidence and developing methods to interpret heterogeneous results, especially when comparing them with extensive homogeneous historical data. Building upon the use of historical data is the developing strategy of virtual control groups. These statistically and digitally derived data sets are expected to complement, supplement and potentially replace the use of concurrent control animals in standard toxicity studies, and may provide a pathway to how more diverse cohorts can be assembled to support regulatory toxicity study endpoints (Golden et al., 2024).

The scientific advantages of utilizing non-naive animals extend beyond simply creating more heterogeneous study populations; these animals can, in some targeted circumstances, provide more clinically relevant models for specific therapeutic areas. For instance, in ophthalmology studies, age-related physiological changes are often a critical factor. Vitreous opacities or changes in vitreous density, which are commonly observed in older human patients, are also present in aged NHPs. Therefore, using older, reused NHPs provides a more translatable model for evaluating therapeutics intended for an aging demographic, an advantage that cannot be replicated in younger naive animals. Similarly, the growing demand for treatments targeting metabolic diseases, such as glucagon-like peptide-1 (GLP-1) agonists for diabetes and obesity, has increased the need for animals exhibiting these conditions to explore mechanistic insights (Zhang et al., 2011). Evidence obtained from large-scale analyses demonstrate that age, environment and individual biological variation can meaningfully influence phenotypes and thereby translational relevance (Jaric et al., 2022). Retaining and then reusing older animals, which may naturally develop age-related metabolic shifts or a propensity for weight gain, can serve as a valuable and more readily available resource for these programs. This strategic selection of non-naive animals with specific acquired characteristics enhances the predictivity of nonclinical studies by more accurately modelling the target patient population, ultimately improving the translatability of the data.

### 4.3 Operational benefits of reuse

The key objectives for increased reuse include ethical and animal welfare considerations, potential scientific benefits in certain contexts as described in Section 4.2, and sustainability. These drivers align with growing pressure on the pharmaceutical industry to minimize animal use, manage development costs, and accelerate timelines to deliver new medicines to patients.

Reusing animals offers a way to mitigate supply chain vulnerabilities by reducing the demand for naive animals. This vulnerability was highlighted during the COVID-19 pandemic, when increased demand for NHPs depleted global breeding stocks and caused significant resource constraints. This NHP demand is further compounded by the increase in emerging modalities such as oligonucleotides, RNA, and gene therapies, for which NHPs are often the most scientifically relevant model due to their unique pharmacology, immune interactions, and limited suitability of alternative species. The consequences of supply shortages can be significant, including reduced availability of sexually mature animals, increased strain on breeding colonies (leading to increased stress levels and infection rates), and even incidents of spurious trading to replenish colonies (USDOJ, 2022). These supply chain issues are not limited to NHPs; other species, such as dogs, also face significant challenges as discussed in Section 3.

Drug development is time intensive and costly, and reuse initiatives that help reduce the time to reach first in human clinical trials will have a positive impact on overall cycle times. Removing the need for animal breeding, transportation, acclimation and training from the list of pre-in-life activities can significantly reduce the study cycle times and increase the speed of drug development. However, this approach also incurs costs associated with extended colony maintenance (including housing, feeding, veterinary care, and personnel), which can vary depending on the duration of the washout and recovery periods.

Where feasible, lowering supply chain costs and shortening study cycle times may contribute to reduced development costs and earlier delivery of urgently needed medicines, although these benefits must be weighed against the ongoing costs of maintaining non-naive colonies.

## 5 Opportunities for increased animal reuse

### 5.1 Current landscape and challenges

Currently, enabling reuse of animals within the pharmaceutical industry is not well coordinated and lacks an industry-wide strategy or synergistic collaboration between sponsor companies and/or CROs. Each company may apply its own reuse process and utilize their own in-house tools, resulting in a fragmented approach that varies by geographical region. Reuse decisions at the scientific team level require a risk-benefit assessment on a case-by-case basis, and while considered routinely at some companies, these assessments may not currently be considered at all by others.

The lack of standardized tools and visibility of available non-naive animals makes it difficult for CROs and Sponsors to facilitate reuse and limits broader implementation at scale.

**FIGURE 2:**
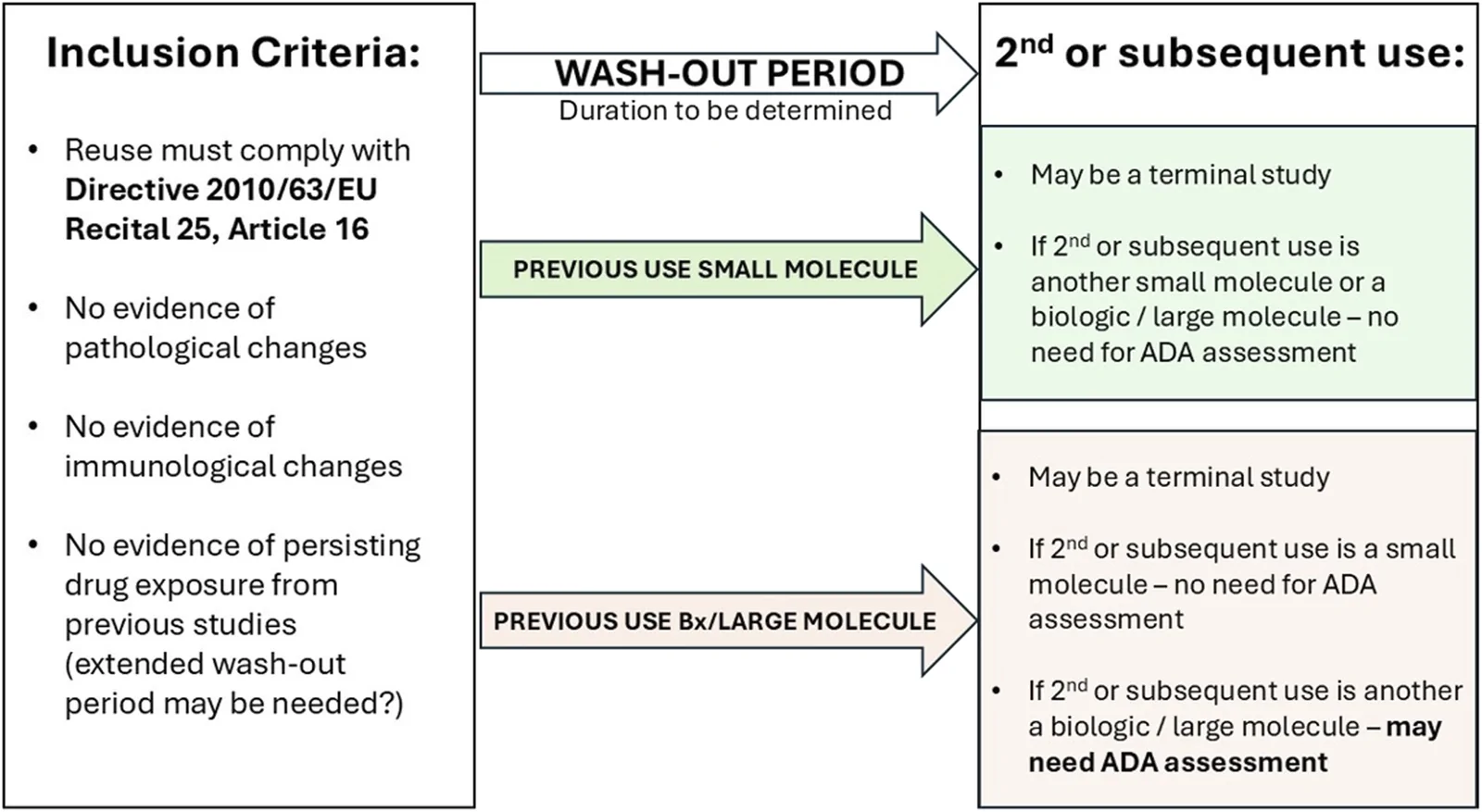
Framework for animal reuse in regulatory studies. This decision tree outlines the necessary inclusion criteria and regulatory considerations for ethical animal reuse. Key requirements include compliance with EU Directive 2010/63/EU, the absence of pathological or immunological changes, and an adequate wash-out period to ensure absence of persisting drug exposure.

Available tools (such as the decision tree and risk matrix shown in Figure 2 and Table 3) are often not standardized or widely shared within the field. As a result, these tools often differ in structure and criteria, making it difficult to achieve alignment across organizations. Consequently, pharma safety assessment teams frequently default to the perceived lowest risk decision of not using non-naive animals. Reasons for rejecting the reuse of non-naive animals include; concerns about long or incomplete washout periods, potential loss of exposure due to ADAs, risk of antibody complex formation, concerns about data variability due to increased heterogeneity, as well as the applicability of historical control data which are typically derived from naive animals. For example, biologically non-naive NHP are frequently difficult to reuse, as sponsors are often unwilling to take the risks and challenges that reusing these animals presents and additionally NHPs are becoming less frequently used in small molecule projects, where reuse might be more easily implemented. Some of the same reasoning is also applied to the reuse assessment of other large animal species, such as dogs and minipigs, although their use in biologics programs is less common. The reuse of rodents, hamsters, and rabbits remains relatively rare for all modalities despite its increased feasibility and is often unexplored by companies.

**TABLE 3:** Risk assessment matrix for anti-drug antibody (ADA) development.

Past exposure	Planned exposure	Risk level	Rationale for risk assessment
Small molecule	Small molecule	Low	ADAs are unlikely to form against small molecules
Small molecule	Large molecule	Low	Initial small molecule exposure is unlikely to generate ADA or prime the immune system for increased response to biologics
Large molecule	Small molecule	Low–Medium	ADAs may have formed from the first biologic molecule, but they are unlikely to affect the second small molecule’s exposure
Large molecule	Large molecule	Medium–High	Potential for cross-reactivity or enhanced clearance; ADAs from the first biologic exposure may impact the second biologic exposure

Concerns about an animal’s prior drug exposure (from the previous study) and the likelihood of having or developing ADAs in the second study that may impact exposure is one of the most frequently cited reasons for not reusing biologically non-naive animals. This is especially relevant to NHP reuse as they are frequently the most scientifically “relevant” species for biologics programs. The decision to exclude reuse based on ADA risk is only relevant to animals having prior exposure to large molecule drugs and that may be included in subsequent studies involving large molecules drug candidates. The risk of ADA impact in the second study depends on the modality of the drug in the second study (Table 3).

The closer the second drug is structurally to the large molecule used in the previous study, the higher the risk of existing ADA impacting the exposure of the second drug.

Other large molecule modalities such as oligonucleotides are smaller and generally less immunogenic than antibodies. When ADAs do form against oligonucleotides, they are often directed against the chemically modified backbone, the sequence itself, or the delivery vehicle. The reuse of biologically non-naive NHP, where the first study was conducted with an antibody drug candidate, should theoretically not have any ADA impact in a subsequent study using an oligonucleotide drug candidate (Grudzinska-Goebel et al., 2025).

### 5.2 Holistic strategies and collaboration

Besides PK/PD and safety pharmacology colonies that exist within pharma or at CROs globally, current reuse of large animal species typically relies on outreach by CROs to individual pharma companies to notify them of available non-naive animals. Following numerous conversations within pharma, a company may decide to use these animals in an upcoming study, which is often a terminal study. A less common approach involves pharma companies proactively identifying non-naive animals at CROs that can be incorporated into future studies or by identifying non-terminal studies in their own planning that could potentially provide a reuse opportunity.

Unless a pharma company has its own established reuse colony, it relies almost entirely on CROs to manage non-naive animals that may have been purchased by the pharma company for a specific study. If the study is non-terminal the animals may go back to stock for further use, however beyond that point the pharma company has limited or no control over the animal’s future unless it has identified another study within its own planning that can reuse these animals. Not all CRO sites have dedicated cohorts of non-naive animals for PK/PD and safety pharmacology that can accommodate other non-naive animals (from non-terminal studies). As a result, space for additional non-naive animals is limited, and the availability of non-naive animals (at preferred partner CROs) rarely matches with the timing of the study needed by other pharma companies.

A related consideration is the applicability of HCD, which are typically derived from naive animal populations. With appropriate washout and recovery, non-naive animals may be considered equivalent or comparable to naive animals, however interpretation of any histological or clinical pathology findings that arise only in the non-naive animals may require additional context (e.g., prior exposure) on a case-by-case basis. In practice, relevant reference and baseline data are often available within sponsor or CRO databases, which can support interpretation and help determine whether data are within expected ranges. For example, following the integration of non-naive animals into a study consisting mainly of naive animals, a notable finding may occur in a single non-naive animal. If the finding is a spontaneous background finding, it may also be present in the HCD and is therefore unlikely to be due to the current or previous drug exposure. If the finding is present only in the high dose group and not in the HCD it is necessary to understand whether it is consistent with the current drug’s mechanism of action.

Alternatively, if all investigations lead to the suggestion that the finding could have been due to a previous drug exposure, this will need to be detailed and explained using a weight of evidence statement to dismiss the findings relevance to the current drug exposure.

Overarching holistic strategies are needed to support a balanced scientific decision-making process that highlights the benefits of reuse rather than focusing solely on its complexities and risks. Future reuse strategies should aim to optimize planning and scheduling, potentially making the use non-naive animals the “default” at the operational level, with the emphasis on the pharma team providing rationale for *not* using non-naive animals, rather than provide a rationale for using them. This approach helps align stakeholders around the goal of achieving ethical animal use while optimizing study conduct to deliver robust and reliable data.

Developing planning collaborations between pharma and CROs will help to incorporate non-naive animals into a more routine strategy. Confidentiality concerns regarding an animal’s previous administration regimen can be overcome through collective standard agreements that allow a limited but informative set of data to be shared post study. Active engagement from all partners would be necessary as the responsibility for coordinating reuse cannot rest with the CRO alone. The optimal scenario would require the pharma company to share an advanced planning forecast with the CRO, identifying studies that could use non-naive animals and for the forecast to match the availability of non-naive animals at the CRO on the required starting dates.

**FIGURE 3:**
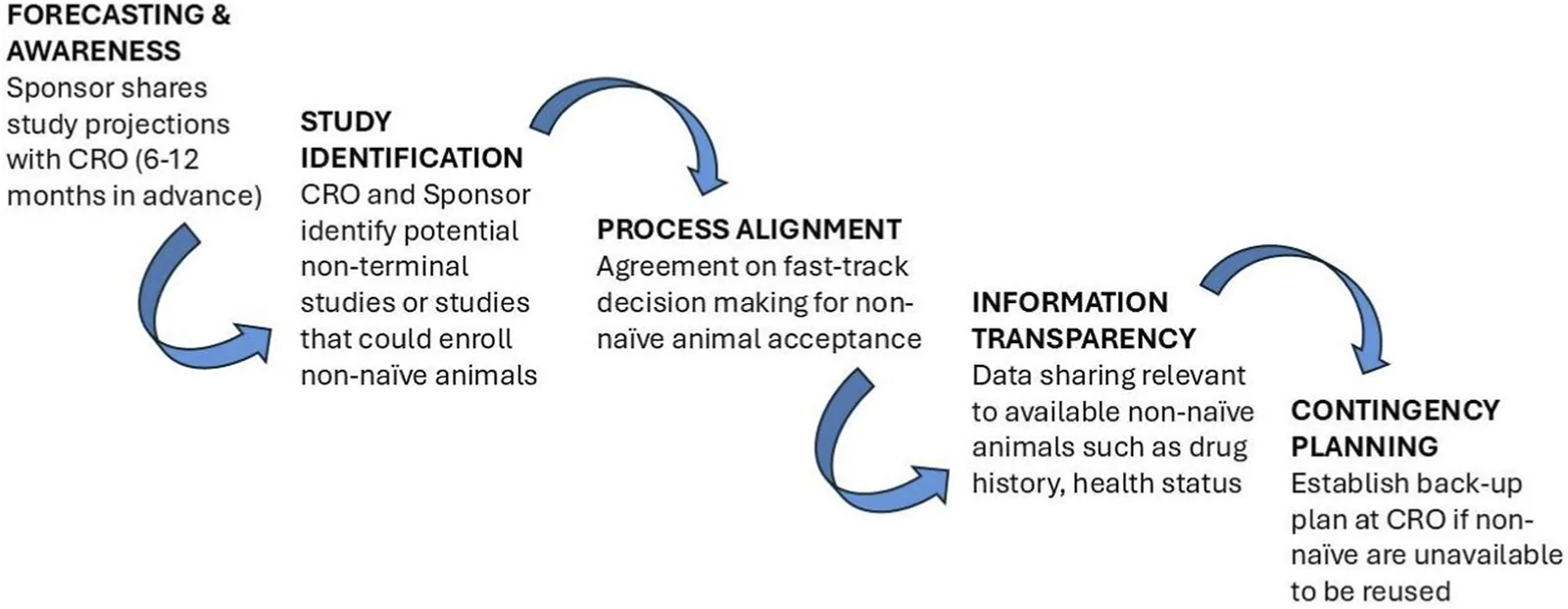
Strategic considerations for the optimization of animal reuse logistics. This flow diagram outlines strategic considerations for optimizing animal reuse through five core phases. By aligning Sponsor projections with CRO non-naïve animal availability 6–12 months in advance and sharing animal data for timely decision-making, both Sponsors and CROs can establish a scalable, integrated system for more efficient enrollment of non-naïve animals.

Considerations and practical steps for this type of forward planning are outlined in Figure 3. This approach is likely to be most effective when multiple pharma companies coordinate their needs across several CRO sites, forming the basis and vision for a potential “cross-pharma reuse consortium”.

### 5.3 New approaches for increasing reuse

Beyond improving decision-making and resource planning, other new approaches are also needed to ensure availability of animals, as the number of non-naive animals available at any specific CRO site at any given time is typically relatively low. Two proposed approaches that could ease this availability challenge are (A) the implementation of multisite *in vivo* studies and (B) increasing the frequency of non-terminal studies (by utilizing biomarkers and a weight-of-evidence approach).

- Multisite *in vivo* studies offer a potential solution to the limited supply of non-naive animals by enrolling smaller cohorts of available non-naive animals at two or more sites. This proposal mirrors the existing human clinical trial standard approach. The advantages of this multi-site nonclinical approach would be to obtain animals that are in short supply, such as sexually mature NHPs from a specific origin, that could be utilized faster without the need to transport the animals to a single test site. In gene therapy programs, multisite approaches may also help identify seronegative animals more efficiently when large numbers of animals must be screened.

Logistically, this approach contains challenges, such as shipping multiple lots of test materials to different sites. However, the costs of extra shipping would likely be negated by saving time, reducing animal costs, and avoiding extra stress in the animals due to transportation.

From a regulatory perspective, this approach is permitted as it would adopt the established principles of a standard multi-site study approach.

While largely untried in the nonclinical field, this strategy could enhance reuse by achieving the generally larger animal numbers required for toxicology studies, compared with the much smaller numbers typically required for PK or safety pharmacology studies where reuse is already established. However, several scientific and operational challenges must also be considered, including the potential need for separate concurrent control groups at each participating site, which may increase total animal numbers (although VCG could be implemented as a solution to avoid this), as well as differences in procedures, housing conditions, analytical methods and staff experience that could introduce variability and complicate interpretation of safety data. For these reasons, multisite designs may be most appropriate for specific, well-defined scenarios rather than as a universal approach.

An additional benefit of this proposal is that multisite involvement may highlight strain or environmental differences, demonstrating that heterogeneity identifies the true range of effects rather than just controlling data variability, which could improve confidence in the translatability of nonclinical safety data (Voelkl et al., 2018).

B. Increasing non-terminal studies via implementation of relevant biomarkers and reducing the need for histopathological endpoints could be an approach that allows greater reuse of animals as it would generally avoid the need for euthanasia. For example, by embracing the advances in safety biomarkers (Table 4), including clinical pathology parameters, physiological biomarkers (e.g., bodyweight, blood pressure, respiratory rate etc.) and omics (such as transcriptomics to identify gene expression changes). Many of these parameters can be obtained from blood, urine, or other biological fluid without the need for necropsy. Histopathology is not a regulatory requirement for all study types and where it is not required, avoiding extensive sampling, processing, and scanning of slides can reduce time, costs and animal use and enable reuse. Histopathology is still required for the majority of Good Laboratory Practice (GLP) compliant toxicology studies to determine target organs and to understand the pathogenesis of observed toxicological effects. However, in early non-GLP studies it may be possible to determine the dose level feasibility or tolerability using a non-terminal weight-of-evidence approach. For certain well-characterized modalities, particularly biologics with known target pathways, non-terminal designs can provide sufficient information on tolerability or early pharmacology without requiring terminal endpoints, enabling decision-making whilst maintaining animal reuse options. Such approaches may also support situations where NAM derived-data can be integrated with in-life biomarker data as part of weight-of-evidence based approach.

**TABLE 4:** Examples of diagnostic biomarkers of organ toxicity and disease.

Organ/disease	Pathological finding	Biomarkers and diagnostic tools
Liver	Hepatotoxicity, drug-induced liver injury	GLDH, TBL, ALP, AST, ALT, miR-122, miR-192
Kidney	Nephrotoxicity, tubular damage	KIM-1, clusterin, creatinine, BUN, GFR effects
Lung	Acute injury	Bronchoalveolar lavage, miRNAs
Heart	Degeneration, necrosis, arrhythmia	Cardiac troponin-1, *In vivo* QT, ECG
Brain	Acute injury	Electrophysiological markers, Functional observation battery
Testis	Drug-induced testicular injury	Circulating miRNAs
Cancer	Breast cancer expression (PDX models)	HER2 expression
Endocrine disease	Blood sugar,long-term glycemic control	Glucose, HbA1c, C-peptide
Inflammatory disease	Systemic inflammation	C-reactive protein
Systemic toxicity	Pain, stress, reduced growth, appetite	Clinical observation, body weight, food consumption

The inherent unpredictability of outcomes in early-stage studies such as dose range finding studies presents challenges for reuse as unexpected adverse findings may exceed mild to moderate severity or prove irreversible, necessitating the exclusion of animals from further use for welfare reasons. This unpredictability is often linked to study design aspects and can be circumvented by carefully considering two main factors. Firstly, as in clinical studies, the dosing of all groups should not generally proceed in parallel. A staggered sequential approach can avoid losing animals at the high dose when the previous dose level may have already shown signs of adversity. Secondly, the rationale for the high dose selection may be based on limit dose or maximum feasible dose criteria, when this has no relevance to the expected clinical dosing. Signs of toxicity can include body weight loss, changes in clinical pathology parameters and clinical observations and should not cause death or severe irreversible suffering.

Several relevant reasons for limiting the dose escalation include; if the systemic exposure in the animal is 50 times higher than the intended human exposure, if the drug’s absorption becomes “saturated” or if the biological effect hits a maximum (saturation of the receptor).

The translatability of nonclinical safety biomarkers to humans continues to improve, supported by advances in digitization, data integration, and use of AI-enabled back-translation approaches. A wide range of qualified biomarkers for detecting organ injury or physiological disturbance already exists, and many of these can be incorporated into early nonclinical studies to provide a weight-of-evidence assessment without the need for necropsy in certain cases. In addition to delivering 3Rs benefits through refinement and enabling non-terminal study designs, biomarker-driven approaches can also generate more clinically relevant data, provide faster decision-making, and reduce the required resources. Table 4 lists some of the commonly used biomarkers categorized by therapeutic area and function that demonstrate strong translatability between animals and humans. Translatability is further enhanced if the nonclinical and clinical correlation exists in the same matrices (e.g., blood, plasma, urine, etc.) and if the effect on the biomarker is related to the pathway of target engagement. Importantly, non-terminal study designs can also help fill the gap between NAMs modelling early-stage disease and whole organism responses by confirming that the early mechanistic signals observed in a NAM are reflected *in vivo*. Non-terminal studies can therefore provide reassurance of NAM relevance without requiring extensive histopathology in appropriate early-phase contexts and without precluding the reuse of animals.

While these biomarkers offer valuable opportunities for non-terminal study designs, it is important to recognize that several of the biomarkers listed in Table 4 are not yet fully validated for regulatory decision-making. For non-validated biomarkers, histopathology remains essential both to confirm adversity and to support biological interpretation. Interpretation can also become more challenging in less standardized study environments, where procedural or environmental variability may influence biomarker readouts. This limitation is even more pronounced for -omics based endpoints, where translational understanding is still developing and additional data are required before such approaches can replace traditional tissue-based assessments. Therefore, biomarker-driven, non-terminal designs should be viewed as complementary tools that can refine and, where appropriate, reduce animal use, rather than replacements for histopathology in studies where tissue evaluation is scientifically or regulatorily required.

## 6 Discussion and conclusion

The reuse of non-naive animals - those that have previously participated in pharmacology and/or toxicity studies for drug development - is an under-utilized, ethically important, and valuable resource for pharmaceutical and biotech companies, particularly when considering their application in nonclinical safety assessment. Table 5 outlines the potential opportunities and challenges of expanding the scope of animal reuse in nonclinical safety assessment studies. While animal reuse has been a standard, regulatory accepted practice for decades, in areas like PK and safety pharmacology studies, its implementation in other areas, such as toxicology, remains limited. This low uptake in regulatory toxicology is driven mainly by logistical, historical, and specific scientific considerations.

**TABLE 5:** Strategic analysis: opportunities and challenges of animal reuse.

Scientific and data quality
Opportunities
Improved translatability: non-naïve animals introduce heterogeneity, reflecting the diversity of human patient populations Reduced stress artifacts: animals acclimatized longer may have lower physiological variability due to transportation and environmental stress, improving pre-dose baseline data Age-related modeling: older, reused animals provide better models for age-related conditions (e.g., ocular or metabolic diseases)
Challenges
Confounding variables: insufficient washout periods may lead to drug-drug interactions or cumulative toxicity Immunological risk: prior exposure to biologics may trigger anti-drug antibodies (ADA), potentially altering the PK of subsequent biological drugs Interpretation difficulty: increased data variability due to heterogeneity can make comparison against historical “homogenous” control data more difficult
Ethics and 3Rs
Opportunities
Reduction: directly decreases the total number of naïve animals bred and transported Refinement: reused animals are often conditioned, trained and socialized for longer, significantly minimizing distress during dosing/handling Minimizing euthanasia: Default reuse prevents the unnecessary euthanasia of healthy, viable animals after a single, low-impact study
Challenges
Cumulative lifetime burden: the impact of multiple studies may exceed the animal’s permitted physiological or psychological burden Social instability: for species such as NHPs, re-housing or re-grouping can disrupt social hierarchies, leading to stress, fighting and weight loss Housing welfare: long-term housing can cause boredom from inactivity or over-stimulation from excessive use; both extremes can potentially trigger atypical behaviors
Operational and supply chain
Opportunities
Sustainability: mitigates global supply vulnerabilities and reduces breeding stock demand Accelerated timelines: eliminates lead-in times for shipping, quarantine, and initial training, potentially speeding up drug development cycles
Challenges
Maintenance costs: long washout or recovery periods incur high costs for husbandry Logistical complexity: aligning the availability of non-naïve animals at a CRO with required study start dates is often complex and challenging Washout management: biologics with long half-lives require lengthy washouts to return to baseline and may still be ADA positive after washout

To address this, we propose adoption of a tiered decision-making framework for animal reuse. This framework will help guide decisions by assessing the nature of the therapeutic drug candidate, the specific biological and scientific endpoints required to evaluate risk, including terminal and non-terminal considerations, and the extent to which other studies in the development package can support the overall safety assessment. Successfully implementing this resource-saving 3Rs approach will require close collaboration between CROs and industry partners.

The ethical considerations of reuse can be viewed from two key perspectives: 1) minimizing avoidable euthanasia of a healthy viable animal and 2) safeguarding animals from excessive cumulative harm when they participate in more than one study. Effective reuse requires careful consideration of both principles, and with appropriate oversight it is possible to satisfy both.

Within the EU and United Kingdom, the standards of husbandry and animal welfare within life science research laboratories are set at a high level through stringent legislative requirements (ASPA, 1986; ETS123, 1986; EU, 2010), which are recognized by international accreditation bodies (e.g., AAALAC) as representing particularly rigorous welfare standards, although other geographical areas rely on locally approved standards. When an animal has achieved its purpose in the study for which it was originally procured and if it continues to be in a healthy and viable condition it can be considered for reuse in further studies expected to impose minimal additional burden (i.e., minimal pain or discomfort). Reuse can be considered for future studies unless the scientific and regulatory requirements of the previous study dictate that histopathological examination must be performed or the procedural burden or adverse effects of the study disqualify the animal from further use. Non-terminal study endpoints can be obtained that allow safety assessment decisions to be made without euthanasia, and this may enable the reuse of animals more frequently and across a greater variety of study types, independent of the animal species. The elucidation of novel therapeutic targets with increased knowledge of the target pathway allows the identification and validation of safety biomarkers in nonclinical species (Zabka et al., 2021). This will be even more critical to provide a robust clinical biomarker monitoring program, to ensure human safety, when NAMs become more accepted as the primary tools for supporting clinical trial applications.

In recent years, events leading to supply chain pressure and increased demands on the ethics and costs of *in vivo* nonclinical safety assessment have changed the focus of the animal reuse discussion. Increased implementation of reuse in nonclinical studies is now seen as a viable proposition, not only for raising ethical standards, but also as a way of sustaining the supply chain and increasing the speed and efficiency of drug development. Other benefits of reuse are understated, such as a way of reducing stressors that can lead to skewed animal data and sub-optimal interpretation of the study’s findings. Holistic reuse strategies or operational level decision-making can enable teams to maximize reuse opportunities that are frequently overlooked. Collaborations between CROs and pharma companies are beginning to take shape and have increased the level and quality of communication around reuse opportunities. However, there is more work to do within the industry to embrace reuse as part of a new, disruptive and revolutionary paradigm shift across all nonclinical species. Ultimately, the replacement of animal use altogether is the long-term vision.

By adapting nonclinical *in vivo* studies to become more aligned with human clinical trial approaches (such as multisite in-life phases and non-terminal study designs), several critical objectives can be achieved. This strategic alignment directly supports the ethical principles of the emerging concept of the 4Rs (Replacement, Reduction, Refinement, and *Responsibility*) in animal research: it enables the generation of robust scientific data sufficient to confer accurate translation to clinical safety in humans, enhances animal welfare through the use of less-stressed animals, and strongly upholds ethical responsibilities by maximizing the value derived from each animal while reducing the overall number needed.

Facilitating this industry-wide shift requires proactive and structured collaboration. Key strategies include deepening CRO-industry partnerships to move beyond transactional relationships toward co-development, enabling shared risk and shared benefit in validating novel, non-terminal study designs. It also involves establishing shared platforms for exchanging historical control data and best practices, which build collective confidence in emerging methods. Achieving this alignment will require sustained commitment across stakeholders; however, the reward has the potential to accelerate development timelines.

Access to well characterized, non-naive animals can shorten lead-in periods, as baseline phenotypic and biomarker data are already established, reducing the need for lengthy acclimation. In addition, non-terminal, biomarker-driven designs can enable faster study read-outs by supporting earlier assessment of tolerability and pharmacodynamic effects, often without reliance on terminal histopathology to trigger go/no-go decisions.

While animal reuse can introduce additional planning considerations, these are typically outweighed by shorter overall study timelines, more rapid access to interpretable data, and faster decision cycles. Collectively, these considerations highlight the value of a consistent, tiered framework to determine when reuse is scientifically appropriate and ethically acceptable, supporting more harmonized decision making across the sector and enabling more efficient progression through early development stages.
