# Supplementary material for: Animal reuse in safety assessment studies: a review of current practices, challenges and 3Rs opportunities
Source: Front Toxicol. 2026 Sep 14;8:1854281. doi: 10.3389/ftox.2026.1854281 (PMC13616529; doi:10.3389/ftox.2026.1854281)
Supplement: Supplementary file 1 [file Supplementaryfile1.pdf]

APPENDIX 1

A case study illustrating reuse of a non-naïve cohort from a non-terminal study into a terminal study.

Background:
Both studies, outlined below, were performed at the same CRO Site requiring no transportation of animals between sites. The advanced planning of the studies enabled the same Sponsor to reuse the animals.

Note - non-naive animals from non-terminal studies can be returned to stock at the CRO and donated to other Sponsors so that they can be reused in other Sponsors studies. This reduces the need for the same Sponsor to have continuous planning at the same CRO site to reuse animals.

Study 1: Non-Terminal Investigative Study Evaluating **Biological Drug X** on Liver Enzymes

Study design and details:
In this investigative study, a cohort of 8 Mauritian naive NHPs, 4M and 4F were utilized in a non-terminal study to evaluate the acute effects of Biologic Drug X on hepatic function following two dose administrations. Key liver enzymes including Alanine Aminotransferase (ALT), Aspartate Aminotransferase (AST), C-Reactive Protein (CRP), and Glutamate Dehydrogenase (GLDH) and pharmacokinetic (PK) and pharmacodynamic (PD) endpoints were evaluated from samples taken via serial 1 mL blood samplings following both administrations. The aim of the study was to inform the project team about the risk for acute liver toxicity via effector function mechanisms. The elevations in liver enzyme parameters were expected to be transient, returning to baseline without inducing clinical toxicity. The study was made non-terminal on the basis that standard clinical chemistry, hematology and some drug-specific non-standard biomarkers in blood, could sufficiently evaluate the risk of acute liver toxicity without the need for histopathology. Importantly, this decision was also supported by the availability of appropriate comparative compounds, which provided a critical reference framework and increased confidence that in-life biomarkers alone would be adequate for assessing the mechanism-related liver liability.

Study Design Table

Group	Main Phase M	Main Phase F
1 (Test)	2	2
2 (Test)	2	2

Post-study:

The animals underwent a defined 6-week recovery and washout period, sufficient to ensure resolution of pharmacological effects and return to baseline clinical parameters. These well-characterized and fully acclimated non-naive NHPs were health checked and approved for study 2. The wash-out /recovery period of 6 weeks between tests was equivalent to the acclimation period of naive animals and longer than the expected half-life of the previous drug. Data acquired from the animals used in Study 1 was fully accessible to the Sponsor. Anti-drug antibody (ADA) evaluation was not considered necessary due to the second study being a small molecule administration study.

Note - biologically non-naive NHPs are rarely re-used in further biological PK/PD studies. Although it is possible to reuse biologically non-naive animals in further biological studies, the risks of subsequent effects on the animals and data integrity require careful consideration. Dialogue between the Sponsor and CRO is necessary to understand the risk of potential ADA generation. Non-terminal PK colonies are generally only established for use with small molecule drugs.

Study 2: 4-Week GLP Toxicology Study with a **small molecule Drug Y**

Study design and details:

The non-naive animals from Study 1 were integrated into Study 2. Instead of using an entirely naive cohort of NHPs for the regulatory GLP Toxicology study, the 8 non-naive NHPs from study 1 were integrated into the larger naive cohort obtained for the GLP study. To mitigate any bias or effects on statistical validity, the non-naive animals were distributed across study groups (shown in **bold** parentheses in the Table below). This combined naive and non-naive cohort approach allowed the 3Rs to be implemented by reducing the total number of naive NHPs required and providing a more comprehensive baseline dataset.

All health records, pre-dose data and available supporting data were checked by the responsible scientists including the on-site Veterinarian, the on-site and Sponsor's Clinical Pathologist, Sponsor Study Monitor and the CRO Study Director.

Study Design Table.

Group	Main Phase M	Main Phase F	Recovery M	Recovery F
1 (Control)	3	3	**(2)**	2
2 (Low dose)	3	1 + **(2)**	-	-
3 (Mid dose)	1 + **(2)**	3	-	-
4 (High dose)	3	3	2	**(2)**

Note - a key consideration in reusing group housed NHP is retaining existing social groupings to reduce individual animal stress. Introducing a new animal into an existing harmonious group is likely to affect the hierarchy of the group leading to fighting and/or over-dominance that can result in skewed or unreliable data. In study 1, the animals were housed in pairs. The main phase groups (low and mid dose) consisted of 3 males and 3 females therefore an additional animal was added to the existing pair in each non-naive group. This was possible by adding a subordinate animal that did not upset the group hierarchy.

Pre-study animal data available to Sponsor prior to study 2 enrolment		
Animals	24 x Naive	8 x Non-naive
Clin Path	1 x pre-dose	2 x pre-dose + data during study 1
Current bodyweights	3-5 kg	2-4 kg
Bodyweight data	Weekly over 2-months	Weekly over 4 months
Age	3-4 years old	2-3 years old
Origin	Mauritian	Mauritian
Sex	12M + 12F	4M + 4F
ADA data	N/A	Yes (*previous drug*)
PK data	N/A	Yes (*previous drug*)
Health screen data	Yes	Yes
Previous drug details	N/A	Yes

Post-study:
The 4-week Toxicology study concluded with a scheduled termination for histopathology.

Note - any histological adverse findings in the 4-week toxicology study that were unique to a non-naive animal would need to be carefully considered to understand whether the finding was test article related, prior drug effects, or a spontaneous background pathology.
